# Single Crystal Sn-Based Halide Perovskites

**DOI:** 10.3390/nano14171444

**Published:** 2024-09-04

**Authors:** Aditya Bhardwaj, Daniela Marongiu, Valeria Demontis, Angelica Simbula, Francesco Quochi, Michele Saba, Andrea Mura, Giovanni Bongiovanni

**Affiliations:** Dipartimento di Fisica, Università degli Studi di Cagliari, I-09042 Monserrato, Italy

**Keywords:** single crystal, Sn perovskites, lead free, halide perovskites, 2D halide perovskites

## Abstract

Sn-based halide perovskites are expected to be the best replacement for toxic lead-based counterparts, owing to their similar ionic radii and the optimal band gap for use in solar cells, as well as their versatile use in light-emitting diodes and photodetection applications. Concerns, however, exist about their stability under ambient conditions, an issue that is exacerbated in polycrystalline films because grain boundaries present large concentrations of defects and act as entrance points for oxygen and water, causing Sn oxidation. A current thriving research area in perovskite materials is the fabrication of perovskite single crystals, promising improved optoelectronic properties due to excellent uniformity, reduced defects, and the absence of grain boundaries. This review summarizes the most recent advances in the fabrication of single crystal Sn-based halide perovskites, with emphasis on synthesis methods, compositional engineering, and formation mechanisms, followed by a discussion of various challenges and appropriate strategies for improving their performance in optoelectronic applications.

## 1. Introduction

Lead-free perovskites appear as ideal materials for improving the prospects of a successful transition to sustainable energy. While perovskite solar cells in general provide the best available combination of efficiency and low cost, lead-free perovskites specifically are more environmentally friendly materials as compared to their lead-based counterparts and could be the next generation materials for revolutionizing solar energy production with sustainable and durable photovoltaic devices [1]. As a matter of fact, the toxicity of the heavy metal component in existing perovskite-based devices restricts their large-scale commercial potential, notwithstanding the remarkable performance of perovskite-based optoelectronics for such a young technology [2]. Tin (Sn) has been projected to be the natural replacement of lead (Pb) due to similar ionic radii (110 pm) vs. (119 pm for Pb) and similar valence electronic configuration ns^2^np^2^, and their use in solar cells has been successfully demonstrated [3,4,5,6,7]. The performances and stability of Sn-based perovskite solar cells are, however, hindered by the instability of Sn^2+^ towards oxidation to Sn^4+^ in ambient conditions, as well as by the abundance of Sn vacancy defects [8], resulting in unwanted doping levels in excess of 10^18^ cm^3^ as well as quick decomposition of the perovskite. Consequently, up to now, Sn-based perovskite solar cells have been markedly lagging in photoconversion efficiencies and operational lifetimes with respect to Pb-based counterparts. Most of the research on halide perovskites focuses on polycrystalline thin films, containing grain boundaries and consequent abundant defects that trap charges and increase nonradiative recombination, hampering device performance. A single crystal approach of fabricating halide perovskites can be an ideal way to improve prospects in optoelectronics. Due to the virtual absence of grain boundaries, single crystals offer high carrier mobilities, low concentrations of trap, and long carrier diffusion lengths [9,10]. Controlled synthesis of single crystals presents very peculiar challenges, and the study of single crystal material properties is typically quite different from its analogue in polycrystalline films. There have been numerous reviews on perovskite single crystals, which focused mainly on the investigation of synthesis processes [11,12] and their usage in solar cells [13], photocatalysis [14] and other optoelectronic applications [6,7,15,16,17,18,19,20,21,22,23]. The stability, density of defects, and material degradation are of paramount importance for Sn-based perovskites, so that the single crystal approach may be even more crucial to develop than in Pb-based counterparts. Even if the fabrication and properties of single-crystal Sn-based perovskites have been addressed within reviews generally covering Sn perovskites or dedicated to single-crystal perovskites, we focus here an entire review on exploring the state of the art and prospects of the intersection between single crystals and Sn perovskites. The overall structure of the review is compiled of four parts. The first part of the review provides a discussion of various fabrication methods and the resulting size of the single crystals, as well as the crystal structure. The second part provides an overview of the special optical and transport properties of Sn single crystals, including defects and doping. In the third part, various applications of Sn-perovskite single crystals in solar cells, photodetectors, X-ray detectors, nonlinear optics, and field effect transistors are elaborated. The last part of the review provides an overall summary of the key challenges that need most attention for boosting the prospects of Sn single crystals for various applications.

## 2. Growth and Fabrication Techniques for Single Crystal Sn-Based Halide Perovskites

The utilization of perovskite single crystals for various optoelectronic applications requires control over the synthesis process, particularly the nucleation rate, to attain a uniform and pure single crystal. However, the controlled synthesis of single crystals, especially those based on Sn-based perovskites, with optimum size and morphology remains complex and challenging. There has been significant advancement in the various techniques used for fabrication of perovskite single crystals within the last few years, with emphasis on improving the crystal quality and purity, ease of process, and reducing growth time. Typically, the synthesis methods for lead-free perovskite single crystals have been mutated from lead-based perovskites, sometimes with additional steps to prevent tin oxidation. This involves using inert atmospheres such as nitrogen or argon to avoid exposure to oxygen and moisture or incorporating reducing agents. Additionally, encapsulation techniques can help stabilize Sn^2+^ by creating a protective barrier. Although the exploration of the role of different types of solvents, both aqueous (such as HI, H_3_PO_2_, etc.) and non-aqueous (DMF, DMSO, GBL, etc.), is still limited, they also play a crucial role in the growth process of single-crystal perovskites. This section summarizes and briefly discusses the various methods used for fabrication of single crystals of Sn halide perovskite.

### 2.1. Temperature Lowering (TL) Method

The temperature-lowering (TL) method or solution temperature lowering is based upon the principle that the solubility of perovskite precursors decreases with decreasing solution temperature. The typical TL method involves three steps: (a) As a first step, the solution for synthesis is mixed and heated at high temperatures to yield a homogenous and clear precursor solution. (b) In the second step, the precursor solution is allowed to cool down to cause precipitation of the seed crystals. (c) In the third step, growth of the seed crystals occurs while the precursor solution cools down. Precise control over the cooling rate allows the growth rate to be managed, and to synthesize large and high-quality crystals [18]. The TL method was used by D Ju et al. [24] to fabricate Sn-based hybrid perovskite single crystals using HI (hydroiodic acid) as solvent. The temperature was lowered from 90 °C to room temperature to obtain single crystals of DMASnI_3_ (where DMA-CH_3_NH_2_CH_3_^+^) with a yellow-colored rod-shaped morphology, which gradually changed to black upon exposure to air. They observed that treatment with deionized water can reversibly change the crystal color from black to yellow; moreover, the crystals showed excellent water stability for more than 16 h. XRD analysis confirmed the formation of an orthorhombic crystal structure. X Jiang et al. [25] grew centimeter-sized 2D mixed Pb and Sn halide perovskite single crystals ((C_8_H_9_F_3_N)_2_Pb_1−x_Sn_x_I_4_ (x = 0, 0.5, 1)) by TL using hydrophobic-conjugated molecules 4-(trifluoromethyl) benzylamine as organic components in a mixed solvent solution of HI-H_3_PO_2_ (hypophosphorous acid). XRD analysis inferred the formation of a 2D-layered structure with a monoclinic system. This growth technique enables effective control over phase purity and crystallinity by minimizing lattice defects, which in turn enhances water stability. However, the process is slow, which can be a drawback for large-scale production, and it demands precise control of the cooling rate.

### 2.2. Top-Seeded Solution Growth (TSSG) Method

**Figure 1:**
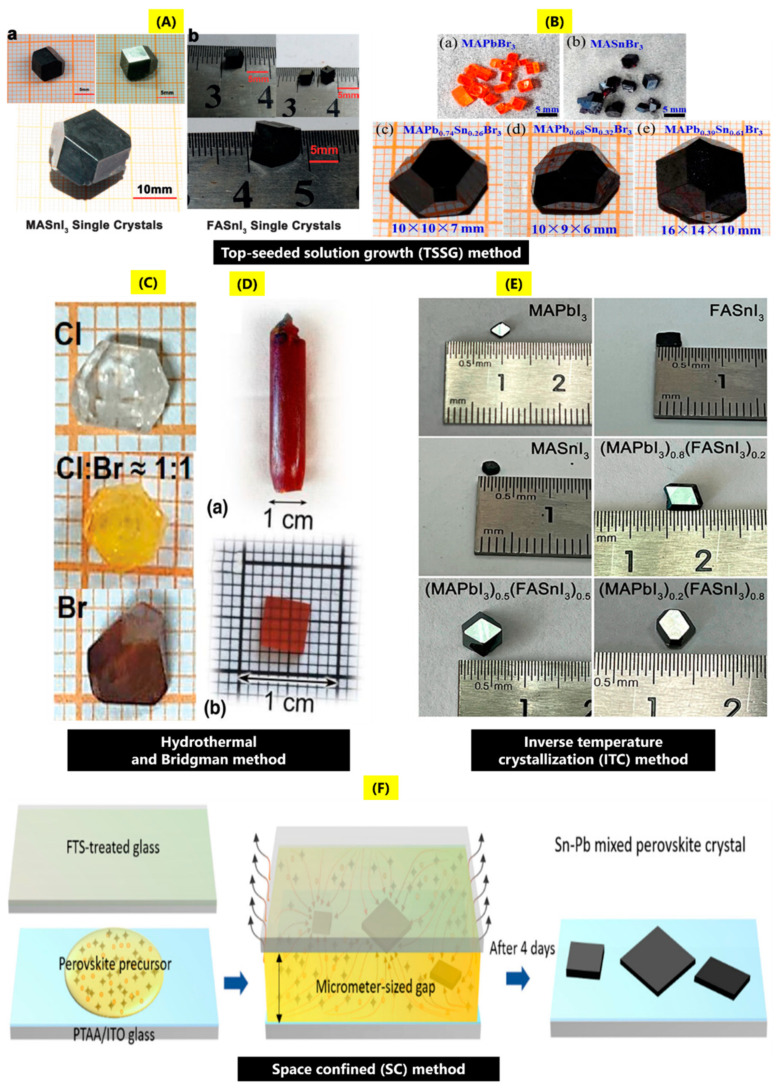
Photographs of the single crystals of Sn-based halide perovskites grown by top-seeded solution growth method: (**A**) (a) MASnI_3_ and (b) FASnI_3_ by optimization of growth conditions (reproduced with permission [26], Copyright Wiley). (**B**) (a–e) Pb-Sn mixed-halide MAPb_x_Sn_1−x_Br_3_ (MA-CH_3_NH_3_) single crystals (reproduced with permission [27], Copyright, ACS Publications), hydrothermal method: (**C**) Color changes of Cs_2_SnCl_6−x_Br_x_ from transparent to yellow to red (reproduced with permission [28], Copyright Wiley). (**D**) Bridgman method for the growth of mixed-halide perovskite single crystals of Cs(Pb_0.75_Sn_0.25_)(Br_1.00_Cl_2.00_) (a) ingot after crystal growth, (b) polished crystal from cleaved part of the ingot (reproduced with permission [31], Copyright Springer Nature. (**E**) Inverse temperature crystallization for the growth of mixed-halides (MAPbI_3_)_x_(FASnI_3_)_1−x_ (x is 0.8, 0.5, 0.2) (reproduced with permission [34], Copyright ACS Publications). (**F**) Space-confined method for the growth of mixed-halide (FASnI_3_)_0.1_(MAPbI_3_)_0.9_ perovskites (reproduced with permission [36], Copyright Royal Society of Chemistry).

The top-seeded solution growth (TSSG) method is based upon optimization of the TL method, wherein the solubility difference of the perovskites in a temperature gradient is the main principle to grow single crystals. The TSSG method was first utilized by Dang and co-workers [26] to obtain single crystals of MASnI_3_ (where MA is CH_3_NH_3_) and FASnI_3_ (where FA is CH(NH_2_)_2_). The synthesis process involves the reaction of formamidinium acetate or methylammonium iodide (MAI) with tin oxide (SnO) in a solvent solution of HI-H_3_PO_2_ at 75 °C, producing spontaneous crystallization of the seed crystals. The crystal growth can be optimized by controlling the temperature and selecting high-quality seed crystals. The fabricated crystals of FASnI_3_ and MASnI_3_ had dimensions of 8 × 6 × 5 mm^3^, 20 × 16 × 10 mm^3^ (Figure 1A(a,b)), and the single crystal XRD measurements confirmed the formation of cubic crystal structure belonging to the Pm3m (number 221) space group with no impurities. The obtained crystals showed good stability for one month when stored in ambient conditions. D Ju et al. [27] used the TSSG method to fabricate mixed Pb/Sn perovskite-MAPb_x_Sn_1−x_Br_3_ (MA-CH_3_NH_3_) single crystals 16 × 14 × 10 mm^3^ in size (Figure 1B(a–e)). The synthesis process in the first step used precursors of SnO, PbO, and methylamine hydrochloride (CH_5_N.HCl) dissolved in HBr-H_3_PO_2_ solvent solution at 75 °C and saturated at 68 °C to obtain a bright yellow-colored solution. In the next step, MAPb_x_Sn_1−x_Br_3_ single crystals were obtained by decreasing the solution temperature from 68 °C to 40 °C with a temperature rate of 60 h/°C, 48 h/°C for temperature reduction from 68 °C to 65 °C to 60 °C. The as-synthesized single crystals showed a cubic perovskite crystal structure belonging to the Pm-3m space group and good stability for more than one month as compared to MASnBr_3_. As an evolution of the TL method, it shares the same advantages and drawbacks. However, by carefully selecting and positioning the seed crystal, the orientation of the resulting crystal can be influenced. To maintain the desired orientation throughout the growth process, it is crucial to precisely control factors such as temperature, solution concentration, and cooling rate.

### 2.3. Hydrothermal and Bridgman Method

Two methods commonly used for the fabrication of Sn single crystals are the hydrothermal method and Bridgman growth. D Ju et al. [24] also explored the synthesis of DMASnI_3_ single crystals by the hydrothermal method, wherein dimethylamine hydrochloride and SnO in 1:1 molar ratios were dissolved in HI and transferred to an autoclave for 8 h at 200 °C. J Zhou et al. [28] utilized the hydrothermal method by reaction of CsCl and SnCl_2_ in a haloid solvent solution with different HCl (hydrochloric) and HBr (hydrobromic) ratios at 453 K (~180 °C) for 36 h to fabricate millimeter-sized transparent to yellow to dark red color variations of Cs_2_SnCl_6−x_Br_x_ single crystals when x is increased from 0 to 1 (Figure 1C). XRD confirmed the cubic phase formation of the single crystals with the shifting of the peaks to lower diffraction angles with increasing content of Br. Xian et al. [29] grew inorganic CsSn_x_Pb_1−x_Br_3_ (0 ≤ x ≤ 1) perovskite single crystals by using precursors, SnO, and solvents, HBr and H_3_PO_2_, and then loading them in an autoclave, and the reaction was carried out for 12 h at 140 °C to obtain black-colored crystals. XRD studies revealed the formation of the orthorhombic crystal structure, and the increased Sn content caused a color change of the single crystals from orange to black. The hydrothermal method operates at relatively low temperatures, reducing the risk of decomposition or oxidation of Sn-based perovskites, and it is scalable for producing bulk crystals. However, the process is sensitive to impurities in the starting materials and environmental conditions, which can impact the quality and reproducibility of the grown crystals. The Bridgman growth method involves high vapor pressures and temperatures, which may impact the chemical stability of organic molecules. This approach is routinely utilized to synthesize large inorganic perovskite single crystals. Typically, raw powder precursors are placed in a sealed quartz ampoule under vacuum, then heated in a furnace to temperatures over the precursor’s melting point. When the melting point is complete, the ampoule is moved to the furnace’s low temperature zone with a fixed speed, and the molten material begins to recrystallize. As the temperature drops, the seed crystals develop and form the ideal crystal structure [18]. At specific temperatures, crystals nucleate near the crucible tip [30]. Valueva et al. [31] recently explored the synthesis of mix halides of Cs(Pb_0.75_Sn_0.25_)(Br_1.00_Cl_2.00_) and fabricated a red-orange ingot by the Bridgman method (Figure 1D(a,b)). The synthesis was carried out by placing a pre-reacted CsPb_0.75_Sn_0.25_BrCl_2_ in a fused silica tube and kept in a four-zone Bridgman furnace. The upper two zones were set at 590 °C, while the lower zone was set at 150 °C, then kept at constant temperature for 12 h, followed by cooling to room temperature. XRD analysis confirmed the formation of cubic crystal structure for the mix halide. The Bridgman method produces large, high-quality single crystals with uniform composition, making it suitable for electronic and optoelectronic applications. However, the high-temperature processing is suitable only for inorganic perovskites. Additionally, the complex setup requires precise control, increasing the cost of the process. 

### 2.4. Inverse Temperature Crystallization (ITC)

Typically, an increase in the solution temperature results in increased solubility of the solute in the precursor solutions. But it was observed by Bakr et al. that for some perovskites, when they are dispersed in specific organic cation solutions, solubility decreased with increasing temperature. Thereby, a new fabrication method called inverse temperature crystallization (ITC) was devised. The typical method involves the utilization of solvents such as GBL (γ-butyrolactone), DMF (N,N-dimethyl formamide), and DMSO (dimethyl sulfoxide) to obtain single crystals of different shapes and sizes [32,33]. Q Li et al. [34] utilized the ITC method to fabricate high-quality (MAPbI_3_)_x_(FASnI_3_)_1−x_ (x is 0.8, 0.5, 0.2) single crystals in hexagonal shape with dimensions of ~5 × 5 mm (Figure 1E). The crystals were made by using stoichiometric combinations of methylammonium iodide (MAI), formamidine iodate (FAI), and formic acid (FAH) with a total concentration of 0.85 M in GVL (1,4-pentanolactone) as solvent at low temperatures of 32 °C, 68 °C, 44 °C, 56 °C, and 80 °C, where (MAPbI_3_)_0.2_(FASnI_3_)_0.8_ grow at 32 °C. The mixed Sn-Pb single crystals show two prominent diffraction peaks at 2θ = 19.8°, 40.24° corresponding to (200) and (400) crystal planes, where the orientation of the crystal is along the (100) direction. ITC produces well-formed, high-quality crystals with low lattice defects, and it offers precise control over nucleation and growth, resulting in high purity, uniform crystals, and reducing unwanted phases. However, ITC requires precise temperature control to avoid oxidation or degradation of the material and is sensitive to environmental factors like humidity and impurities.

### 2.5. Space-Confined (SC) Method 

The space-confined method (SC) has been found to be a suitable fabrication process to control the thickness of the perovskite single crystals. In this method, typically the precursor solutions are geometrically confined between two substrates; altering the geometric space results in adjusting the thickness of the crystals. It can also be combined with other fabrication techniques such as TL, TSSG, ITC, etc., to obtain single crystals with controlled thickness and geometry [35]. Z Chang et al. [36] reported the synthesis of (FASnI_3_)_0.1_(MAPbI_3_)_0.9_ single crystals by the low-temperature SC method (Figure 1F). In the synthesis process, in a N_2_-filled glove box, a volume of 5 μL of (FASnI_3_)_0.1_(MAPbI_3_)_0.9_ solution (mixing of FASnI_3_ and MAPbI_3_ in a 1:9 stoichiometric ratio for 1h at 37 °C, where individual FASnI_3_, MAPbI_3_ solutions were prepared from FAI+SnI_2_+10 mol% SnF_2_ in 200 µL PC/GBL solvent and MAI + PbI_2_ + 3.5 mol% Pb(SCN)_2_ in 300 µL PC/GBL solvent was dropped onto PTAA/ITO substrate that had been heated to solution temperature and covered with a glass substrate coated with FTS (trichloro (1H,1H,2H,2H-perfluorooctyl) silane). The two substrates were then placed on a hotplate at a temperature of 25–30 °C to promote nucleation and growth after being clipped together. To optimize the thickness of the crystal, the applied force of the clip is adjusted to manage the gap between the upper and lower substrates. In the last step, the two substrates were finally separated to produce thin single crystals of thickness ~1.4 μm on the PTAA/ITO. The substrate was then placed on the hot plate to gradually cool to ambient temperature and further remove residual solvent. XRD analysis confirmed formation of the tetragonal lattice with two major diffraction peaks of the (200) and (400) planes. Confined crystal growth allows for precise control over the thickness and orientation of crystals, while the controlled environment minimizes defects and unwanted phases. Currently, this technique is unique in its ability to produce single-crystal thin films that can be integrated into devices such as solar cells and LEDs. However, the confinement may restrict the maximum size of the films, which could be a limitation for applications requiring larger surfaces.

### 2.6. Synthesis Methods for 2D Sn-Based Halide Perovskite Single Crystals 

**Figure 2:**
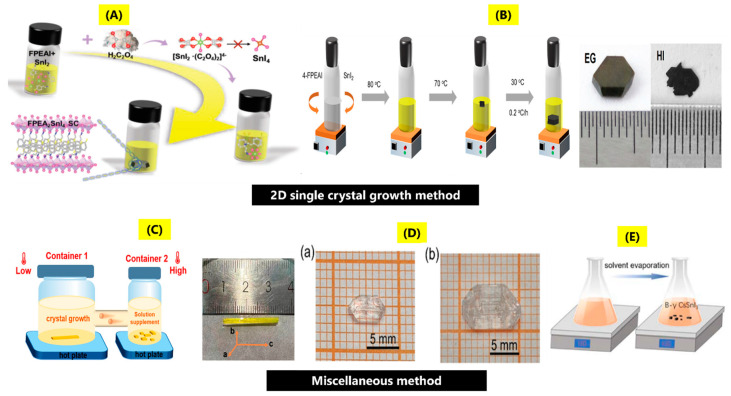
(**A**) Reductant engineering method using oxalic acid for the growth of FPEA_2_SnI_4_ single crystals (reproduced with permission [37], Copyright Wiley). (**B**) Ethylene glycol and HI-assisted modified temperature lowering method for fabrication of 2D (4-FPEA)_2_SnI_4_ tin halide perovskite single crystals (reproduced with permission [38], Copyright ACS Publications). (**C**) Synthesis process for fabrication of 1D perovskite single crystal-MDASn_2_I_6_ and its corresponding image and dimensions (reproduced with permission [49], Copyright ACS Publications). (**D**) Single crystals of (a) (R/S-*α*-PEA)SnCl_3_ and (b) (R/S-*α*-PEA)SnBr_3_ of sizes-6 mm, 10 mm by bottom seeded solution growth (reproduced with permission [50], Copyright Wiley). (**E**) Single crystals of B-γ CsSnI_3_ by solvent volatilization method (reproduced with permission [51], Copyright Royal Society of Chemistry).

M Li et al. [37] used a reductant engineering approach by using H_2_C_2_O_4_ (oxalic acid) as a reductant to synthesize FPEA_2_SnI_4_ (p-fluorophenethylamine tin iodide) single crystals (Figure 2A). During the synthesis process, oxalic acid acts as a bidentate ligand, assisting in the formation of SnI_2_ (stannous iodide). As the temperature decreases, the coordinated oxygen atoms are slowly replaced by coordinating I ions to form stable [SnI_6_]^4−^ metal octahedron. This is in conjunction with the organic cations, which results in a 2D-layered perovskite structure. XRD measurement revealed diffraction peaks corresponding to (00k) planes of 2D perovskite single crystals. C Yan et al. [38] used a modified temperature-lowering method to fabricate (4-FPEA)_2_SnI_4_ single crystals. Ethylene glycol (EG) was explored to fabricate 2D Sn-based single crystals (Figure 2B). The strong coordination between EG and SnI_2_ helps in the slow growth of the perovskite single crystals with dimensions of 5 × 5 × 1 mm^3^. This study tried to grow (4-FPEA)_2_SnI_4_ single crystals by using 4-FPEA (4-fluorophenethylammonium) as an organic cation using HI and EG as solvents, wherein better control over crystal growth could be achieved by EG as solvent. Black-colored 2D perovskite single crystals were obtained, and XRD analysis revealed the single crystal belongs to the P2_1_/C space group. The synthesis method can also be used to grow other 2D single crystal perovskites with different organic molecules, such as (3-FPEA)_2_SnI_4_, (2-FPEA)_2_SnI_4_, and (PEA)_2_SnI_4_. Y li et al. [39] used the slow cooling method to fabricate 2D Ruddlesden–Popper tin halide perovskite single crystals. The changes in the precursor concentrations of BAI (butylammonium iodide), PEAI (phenethylammonium iodide), MAI (methylammonium iodide), and SnI_2_ (tin (II) iodide) in solvents composed of HI and H_3_PO_2_ resulted in various single crystals such as (BA)_2_MA_n−1_Sn_n_I_3n+1_ (n = 1 to 4), (PEA)_2_MA_n−1_Sn_n_I_3n+1_ (PEA^+^: phenethylammonium and n = 1, 2), (2T)_2_MA_n−1_Sn_n_I_3n+1_ (n = 1 and 2) (2T^+^: bithiophenylethylammonium), and (3T)_2_MA_n−1_Sn_n_I_3n+1_ (n = 1 and 2) {3T^+^: 2-([2,2′:5′,2″-terthiophen]-5-yl)ethan-1-aminium}. From XRD analysis, the crystal structure for (BA)_2_MA_n−1_Sn_n_I_3n+1_ (n = 1 to 4) was found to be orthorhombic, while for (2T)_2_MA_n−1_Sn_n_I_3n+1_ (n = 1 and 2), (3T)_2_MA_n−1_Sn_n_I_3n+1_ (n = 1 and 2) single crystals, monoclinic, and triclinic crystal structures were observed. Also, the interlayer distance was found to increase when the spacer ligand changes to 3T^+^ from PEA^+^. Y li et al. [40] synthesized 2D Sn halide perovskite single crystals: (PEA)_2_MA_n-1_Sn_n_I_3n+1_ (n = 1–4) by using precursors, PEAI, MAI, and SnI_2_, in solvents HI and H_3_PO_2_, with heating to dissolve precursors and slow cooling at room temperature. (PEA)_2_MA_n-1_Pb_n_I_3n+1_ (n = 1–4) crystals have similar XRD patterns compared to those 2D tin counterparts. Few weak XRD impurity peaks were observed in (PEA)_2_MA_3_Sn_4_I_13_ due to difficulty in controlling the phase purity as n value increased. In. H-Park et al. [41] studied the single crystals of 2D tin iodide hybrid perovskite-[A_2_B_(n-1)_Sn_n_I_(3n+1)_] (n = 1 and 2) by reaction with precursors of tin iodide, 2-(4-stilbenyl) ethanammonium iodide, and 2-(4-(3-fluoro) stilbenyl) ethanammonium iodide. The different precursors resulted in different Sn iodide single crystals: (BA)_2_SnI_4_, (PEA)_2_SnI_4_, (FSA)_2_SnI_4_, (FSA)_2_(MA)Sn_2_I_7_. The reaction of the precursors at 90 °C in hydroiodic acid and ethanol, followed by slow cooling, resulted in dark-red plate-shaped single crystals. XRD analysis confirmed triclinic structure belonging to the P-1 space group. G Ding et al. [42] explored the synthesis of 2D Ruddlesden–Popper (TEA)_2_(MA)_n-1_Sn_n_I_3n+1_ (n = 1, 2, TEA, MA is 2-thiophenethylamine, methylamine) tin iodide single crystals. The single crystals were fabricated by the solution temperature cooling method; ethanol addition was used for controlling the solubility of the perovskite precursor. The synthesis was performed using the mixing of two solutions: solution (i) 2 mmol TEA + 1.5 mL HI + 0.5 mL ethanol, solution; mixing resulted in yellow TEAI solution; (ii) 1 mmol SnO + 1.5 mL HI + 0.4 mL H_3_PO_2_, mixing resulted in yellow SnI_2_ solution. Thereafter, two solutions were dissolved for 3 min at 90 °C; a dark-green irregular powder was obtained after mixing, due to large supersaturation of the precursors; then, 1.5 mL of ethanol was added (excellent solubility for TEAI, SnI_2_), and the solution was heated in an oil bath at 70 °C for another 40 min. Cooling resulted in centimeter-sized single crystals. XRD measurements confirmed the formation of triclinic crystal structure belonging to the P-1 space group. C Liao et al. [43] reported the fabrication of various Dion−Jacobson (DJ) tin halide perovskite single crystals denoted as 3AMPSnI_4_, 4AMPSnI_4_, 3AMPYSnI_4_, and 4AMPYSnI_4_ ((AMP is (aminomethyl)-piperidinium, AMPY is (aminomethyl)pyridinium)) by antisolvent vapor-assisted crystallization. The synthesis process involved a 1:1 molar ratio of AMPI_2_/AMPYI_2_, mixed in GBL. The reaction was carried out for 1 h. Thereafter, the solution was filtered, transferred to a vial, and then DCM (dichloromethane) was used as solvent. The solution was kept for 3 days to obtain tin single crystals. The size of the single crystals was 0.16 × 0.12 × 0.04 mm^3^, 0.12 × 0.11 × 0.10 mm^3^, 0.08 × 0.07 × 0.03 mm^3^, 0.05 × 0.03 × 0.02 mm^3^ for 3AMPSnI_4_, 4AMPSnI_4_, 3AMPYSnI_4_, and 4AMPYSnI_4_. XRD analysis revealed that 3AMPSnI_4_ has a tetragonal structure, space group P4, while 4AMPSnI_4_, 3AMPYSnI_4_, and 4AMPYSnI_4_ have orthorhombic crystal structures, space groups Pca2_1_, Pnma, and Pmmn. K Li et al. [44] reported the synthesis of multilayered tin-based perovskite single crystals of (BA)_2_(EA)_2_Sn_3_Br_10_ (where BA and EA are butylammonium, ethylammonium) by temperature cooling method. The synthesis process involved first mixing HBr and H_3_PO_2_ (4 mL each) and adding them to 0.5 g SnO. In the next step, n-butylamine and ethylamine solvent solutions are slowly added and stirred for 30 min to obtain a uniform solution. After this, the solution is transferred to an oven and cooled at a rate of 2 °C/day. After cooling, light green crystals are formed. XRD revealed the formation of a cubic crystalline structure belonging to the space group Cmc2_1_. R Zhang et al. [45] explored the synthesis of PEA_2_SnBr_4_ single crystals by the cooling-induced crystallization method. In the typical process, 1 mmol PEABr (C_6_H_5_CH_2_CH_2_NH_3_Br), 0.5 mmol SnBr_2_ in 3 mL of HBr, H_3_PO_2_, then heating solution to 120 °C for 1h; thereafter, the solution was allowed to cool to room temperature, washing with ethanol, and vacuum drying for 12 h at 60 °C yielded the single crystals. Additionally, bismuth-doped single crystals of PEA_2_Sn_1−x_Bi_x_Br_4_ were also synthesized by the addition of BiBr_3_ during synthesis. In the case of PEA_2_SnBr_4_, yellow flake-like crystals, and in the case of Bi doping, black crystals were formed. There was no observation of shifts in the XRD diffraction peaks when the bismuth concentration changed from 1% to 20%; only some change in intensity of diffraction peaks was seen, which could be due to similar radii with tin.

### 2.7. Miscellaneous Synthesis Methods

This section will discuss various synthesis methods that have been explored for the fabrication of Sn perovskite single crystals. A Mhiri et al. [46] reported the synthesis of (TMA)_2_SnBr_6_ (TMA: tetramethylammonium) by the slow evaporation technique using a stoichiometric ratio of SnBr_2_ and [(CH_3_)_4_N] Br in hydrobromic acid with cubic crystal structure, space group Fm3m. Y Dang et al. [47] reported the synthesis of NH(CH_3_)_3_SnCl_3_ and NH(CH_3_)_3_SnBr_3_ single crystals by bottom seeded solution growth. The synthesis process for NH(CH_3_)_3_SnCl_3_ began by dissolving 0.10 mol of SnCl_2_.2H_2_O + 0.10 mol of NH(CH_3_)_3_Cl in 150 mL HCl + 100 mL H_3_PO_2_ solvent solution and mixing to obtain a transparent solution; saturation of the solution at 54 °C for 1 month yielded the single crystals. For the case of NH(CH_3_)_3_SnBr_3_, 0.05 mol SnO + 0.05 mol NH(CH_3_)_3_Cl mixed and dissolved in 200 mL HBr + 50 mL H_3_PO_2_ solvents, saturating the solution at 56 °C for 1 month yielded the single crystals. The sizes of NH(CH_3_)_3_SnBr_3_, NH(CH_3_)_3_SnCl_3_ single crystals were found to be 8 × 6 × 4 mm^3^ and 13 × 8 × 6 mm^3^. The XRD for NH(CH_3_)_3_SnCl_3_ and NH(CH_3_)_3_SnBr_3_ single crystals confirmed the formation of orthorhombic crystal structures belonging to space groups Cmc2_1_ and Pna2_1_. Y Wu et al. [48] explored the synthesis of CH_3_NH_3_SnBr_3_ single crystals, red in color, by volatilizing the solution (1 mmol tin bromide (SnBr_2_) and methylammonium bromide (MABr) in 1 mL of dimethylfumarate (DMF) solvent at room temperature. The XRD analysis confirmed the formation of tetragonal crystal structure. Y Niu et al. [49] reported the fabrication of a non-centrosymmetric 1D perovskite single crystal by selecting organic diamine (Figure 2C). Typically, 4,4′-methylenedianiline (MDA) is used as organic diamine to synthesize MDASn_2_I_6_ perovskite single crystals. These crystals were grown in a saturated acid solution at a constant temperature with continuous solute feeding, wherein container 2, used for continuous solute feeding, was kept at a relatively high temperature, while container 1, used for crystal growth, was kept at a relatively low temperature. The synthesized crystals were rod-shaped and bright yellow colored, having dimensions of 24 × 2 × 2 mm^3^ with an orthorhombic crystal structure. K Tao et al. [50] used combination of chiral organic ions R- and S-*α*-phenylethylammonium (R/S-*α*-PEA^+^) and tin (II) halide inorganic frameworks to obtain two pairs of chiral perovskites, namely (R/S-*α*-PEA)SnCl_3_ and (R/S-*α*-PEA)SnBr_3_, which form large single crystals with dimensions of ~6, 10 mm, that are transparent in nature. The single crystals of (R/S-*α*-PEA)SnCl_3_ were obtained by the bottom seeded solution growth method, in the first step, (R/S-α-PEA). HCl was obtained by the reaction of 3 mL R/S-α-PEA with 3 mL ethanol, followed by the addition of 4 mL HCl in an ice bath. After mixing, the solution was heated at 100 °C for solvent evaporation, heating was closed when the solid phase appeared, and cooling resulted in obtaining white powder. In the second step, SnCl_2_, white powder of (R/S-α-PEA). HCl and H_3_PO_2_ were reacted to obtain single crystals. The single crystals of (R/S-α-PEA)SnBr_3_ were obtained by reaction of 2 mmol SnO, 2mL HBr, R/S-α-PEA, and H_3_PO_2_ in an ice bath. Large crystals of (R/S-α-PEA)SnCl_3_ and (R/S-α-PEA)SnBr_3_ can be obtained by heating at 60 °C, 95 °C, followed by slow cooling to room temperature. XRD analysis confirmed them to belong to the P2_1_2_1_2_1_ chiral space group (Figure 2D(a,b)). In these single crystals, [SnX_3_]^−^ units are connected to form 1D chains, while the stereochemically active lone pair 5s^2^ electrons in Sn^2+^ make the [SnX_6_] octahedra within inorganic frameworks to have high distortion as compared to hybrid halides. Q Sun et al. [51] used the heat-activated solvent volatilization method for 12 h at 130 °C to obtain B-γ CsSnI_3_ single crystals (Figure 2E). The single crystal was found to be cuboid in shape, black in color with dimensions 12 × 7 × 3 mm^3^, belonging to the orthorhombic crystal structure, space group Pnma. 

The development of high-quality hybrid perovskite single crystals is a complicated process that still requires research efforts to gain complete control and competence. In particular, the growth of tin-based perovskite single crystals is more challenging with respect to their lead-based counterparts due to the fast oxidation of Sn^2+^. So far, various synthesis methods have been proposed to synthesize Sn-based halide perovskites. Temperature lowering (TL), top-seeded solution growth (TSSG), and inverse temperature crystallization (ITC) have been demonstrated as successful techniques to grow large bulk Sn-based single crystals. These techniques, however, still require optimization, especially aiming at reducing the reaction times, and they do not allow to control the crystal thickness. Very recently, the space-confined method has been employed to grow single crystals with thickness below a few micrometers. However, this approach requires further investigation to overcome the present limitation on the lateral size of the crystal, which is limited to some hundreds of microns and represents an obstacle for large area application, such as solar cells. At present, the full understanding of the physical mechanisms governing crystal growth, which is necessary to reach full control over the growth parameter, is still immature. Further research effort needs to be dedicated to investigating the role of various types of solvents, reaction parameters (temperature, growth rate, etc.), substrate engineering, and passivation strategies to prevent Sn oxidation and to fabricate high-quality Sn single crystals.

## 3. Charge Transport Properties of Single Crystals of Sn-Based Halide Perovskites

### 3.1. Optical Properties

**Figure 3:**
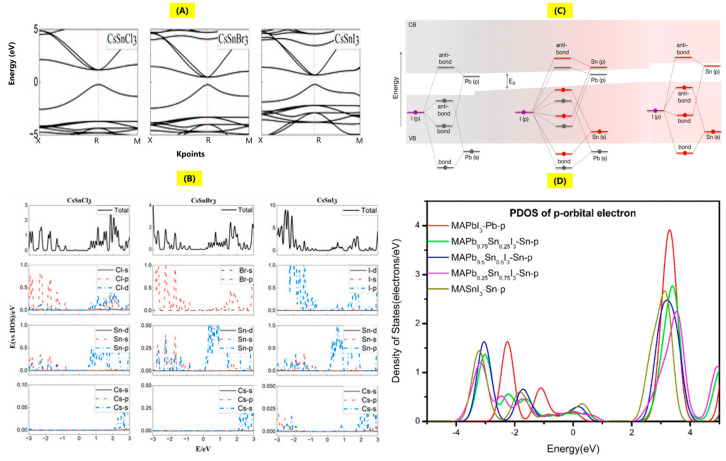
(**A**,**B**) Energy band structures and DOS of CsSnCl_3_, CsSnBr_3_, CsSnI_3_ (reproduced with permission [55], Copyright MDPI). (**C**) Band-bowing in mixed-halide perovskites of MA(Pb_1−x_Sn_x_)I_3_ (reproduced with permission [57], Copyright ACS). (**D**) Partial density of states of p-orbital electron in MASn_a_Pb_1-a_I_3_ mixed-halide perovskites (a-0, 0.25, 0.5, 0.75, 1) (reproduced with permission [58], Copyright Elsevier).

For effective utilization of the perovskite materials in various optoelectronic applications, the essential requirements are a tunable band gap across a wide range and efficient light emission. Information from absorption spectra about the band structure, density of states, and from the photoluminescence (PL) spectra about the photoluminescence peak position, nature of emission, full width half maximum, defects, and photoluminescence yield are among the essential optical properties for successful utilization of perovskite single crystals in optoelectronic applications. There is another parameter called the charge carrier lifetime (τ) that also plays an important role in optical properties, studied by various measurement techniques such as time-resolved photoluminescence (TRPL) spectroscopy, transient absorption, and transient photovoltage decay [19]. TRPL is used to measure the electron–hole radiative recombination after the absorption of a short excitation pulse, which is done by time-correlated single photon counting, wherein the material to be analyzed is periodically excited by a pulsed laser, then estimating the emission at the requisite wavelength [52]. The optical properties in lead-based perovskites are strongly dependent on the [PbX_6_]^4−^ octahedron, and replacement of Pb with Sn causes a red shift in the emission characteristics as well as a band gap reduction [53]. Sn is projected to be the ideal replacement for Pb due to the analogous ns^2^np^2^ electronic configuration, high absorption coefficient, and charge carrier mobility [54]. To discuss the effect of optical absorption and emission measurements for Sn single crystals, it is first necessary to understand the origin of band structure in Sn halide perovskites. We will first discuss various theoretical calculations to have an understanding of the band structure and DOS of Sn-based halide perovskites, followed by experimental studies. Based upon the Heyd–Scuseria–Ernzerhoft–HSE06 hybrid functional electronic structure calculations, the energy band structures of CsSnCl_3_, CsSnBr_3_, and CsSnI_3_ are as shown in Figure 3A [55]. Both Pb and Sn have direct band gaps with similar band order; based upon the increased ion radius from chlorine to iodine, the band gap is found to decrease. Another important parameter that affects the optoelectronic properties is the density of states (DOS) distribution, as it shows the influence of various material compositions of elements on energy bands as well as charge carrier distribution. The calculated DOS distribution of CsSnCl_3_, CsSnBr_3_, and CsSnI_3_ is shown in Figure 3B. It is observed that the valence band maximum (VBM) originates from the p orbitals of the halides (Cl, Br, and I) with minor contributions from s orbitals of Sn or Pb, while the conduction band maximum (CBM) originates from the p orbitals of Sn or Pb, with a minor fraction of p and d orbitals of halides (Cl, Br, and I) [55]. Typically, it has been observed that the band gap is mainly influenced by Sn or Pb as well as halides (Cl, Br, and I), and there is much less significant influence from the Cs cation [56]. The mix halides of Pb and Sn, for example, CH_3_NH_3_(Pb_1−x_Sn_x_)I_3_ perovskites, also cause significant variation in the band structure as well as observation of band bowing, as shown in Figure 3C. The band edges of MAPbI_3_, corresponding to Pb states, are strongly bent in comparison to the band edges of MASnI_3_, corresponding to Sn-s and Sn-p orbitals, resulting in band offsets, as seen from Figure 3C. For mixed-halide perovskites, VBM originates from Sn-s and I-p orbitals and the CBM originates from Pb-p and I-p orbitals; therefore, there is observation of band gap bowing, and the band gap is lowered as compared to perovskites based on pure Pb and Sn, a counterintuitive effect stemming from the differences in energies of s and p orbitals of Sn and Pb [57]. DFT studies of mixed Pb-Sn perovskites also indicate that the band gap decreases from 1.51 eV to 1.30 eV when the Sn ratio is changed from 0 to 100%. From the partial density of states (PDOS) calculations of Sn (Figure 3D), it is noted that as Sn content is increased, the contribution of Sn 4p electrons to the bottom of the conduction band (CB) is also increased while the energy of the p-orbital decreases [58]. 

Table 1 illustrates the typical band gap values of Sn single crystals. The optical measurements of single crystals of MAPb_x_Sn_1−x_Br_3_ revealed that the spin–orbit coupling, and structural distortions with increasing Sn concentration can also lead to shifts of the absorbance and photoluminescence peaks [27]. The photoluminescence studies on 2D layered Sn-based single crystals (C_8_H_9_F_3_N)_2_Pb_1−x_Sn_x_I_4_ (x-0, 0.5, 1) revealed the origin of the PL peaks to self-trapped excitons, due to distorted halide octahedra originating from the size mismatch between inorganic and organic components [25]. Studies on Bi-doped 2D PEA_2_SnBr_4_ (0 to 20%) single crystals have also revealed the variation in photoluminescence due to self-trapped excitons [45]. When large cations are not employed and therefore self-trapping does not occur, the bandgap value of Sn-based perovskites is lower and thus better suited for photovoltaics than the band gap of corresponding Pb-based compounds. The detailed balance limit indicates that when illuminated with the AM 1.5 solar spectrum, single junction solar cells with ~1.1 eV bandgap energy, the value for MASnI_3_, can achieve a photoconversion efficiency of ~30% [59]. The findings of the band gap from Table 1 illustrate that the single crystals of CsSnI_3_, FASnI_3_, and MASnI_3_ are ideal candidates for utilization in perovskite solar cell fabrications, extending the absorption in the infrared region, while the wide band gap single crystals of 2D and 1D tin perovskites can find use in nonlinear optical switching, amplified emissions, discussed in Section 4.

It can be concluded that the intrinsic optical properties of Sn-based perovskite single crystals are even superior to the Pb-based counterparts. The bandgap is lowered when Sn replaces Pb. making Sn perovskites better suited to absorb sunlight and producing larger currents in solar cells. The band structures of Sn and Pb-based perovskites are similar, and thus are the absorption coefficients, typically more than 10^4^ cm^−1^ across the whole visible spectrum, allowing submicron layers to absorb virtually all incoming light. Such properties have been well established also in single-crystal Sn perovskite materials.

### 3.2. Electrical Properties

The successful utilization of Sn-based perovskite materials for optoelectronic applications is directly related to the charge transport parameters: charge carrier mobility (μ), charge carrier lifetime (τ), and charge carrier diffusion length (L_D_) [19]. The charge carrier mobility helps to identify the average drift velocity of charge carriers under the presence of an electric field, denoted by the equation: μ = qτ/m*, where m* is the effective mass of the charge carrier, q is the electronic charge, and τ is the relaxation time of the charge carriers. The charge carrier mobility can be investigated by the Hall effect measurements, space charge limited current (SCLC), time of flight (TOF) measurements [62]. An equally important parameter is the charge carrier lifetime, which is strongly dependent on the quality of the single crystal fabricated. Typically, excitation by photons results in the generation of excess electron–hole pairs; recombination of electron and hole results in the emission of another photon; the overall process is called radiative recombination. But due to the presence of traps, there is also trap-induced nonradiative recombination, which can cause changes in τ and hamper the optoelectronic properties of the perovskite. The investigation of the electrical properties of single crystals can provide direct evaluation of the charge carrier mobilities due to the uniform structure with no grain boundaries. Concerning the charge transport properties of single crystals of CsSnI_3_ [60] grown by the Bridgman method, an increase in the electrical conductivity from 16 to 208 S/cm was observed over six heating cycles at room temperature. Hall effect measurements revealed low carrier concentration <10^17^ cm^−3^, p-type conductivity, and mobility of 585 cm^2^/Vs. The mobility values were in close agreement to the GaAs and InP semiconductors with mobilities of 400 and 700 cm^2^/Vs. The Hall effect studies of top seeded solution growth MASnI_3_ single crystal at room temperature indicated a p-type semiconductor behavior, whereas FASnI_3_ shows n-type semiconductor behavior. The trap densities were found to be of the order ~10^11^/cm^3^ [26]. The study of MAPb_x_Sn_1−x_Br_3_ single crystals grown by top seeded solution growth, by Hall effect measurements observed n-type semiconductor behavior, found the carrier mobility of MAPb_0.68_Sn_0.32_Br_3_ and MAPb_0.39_Sn_0.61_Br_3_ to be 19.61, 32 cm^2^/Vs at 200 KHz, while the trap density and carrier concentration were 6.47 × 10^11^, 5.61 × 10^11^ cm^−3^, and 10^11^ cm^−3^ [27]. Based on the SCLC measurements, the charge carrier mobility for DMASnI_3_ (DMA-CH_3_NH_2_CH_3_^+^) single crystals was found to be 4.75 cm^2^/Vs, trap density 10^11^ cm^−3^, upon increasing the frequency from 10 KHz to 1000 KHz, mobility increased to 11.82 cm^2^/Vs, whereas resistivity measurement by the Hall effect was found to be of the order ~10^7^ Ω cm [24]. The carrier mobility of CsSn_0.5_Pb_0.5_Br_3_ single crystals estimated by SCLC was found to be 1.3 × 10^3^ cm^2^/Vs, much higher than in CsPbBr_3_-15 cm^2^/Vs, with a corresponding trap density of 1.19 × 10^11^ cm^−3^ [29]. The balanced distribution of Sn-Pb atoms in CsSn_0.5_Pb_0.5_Br_3_ results in improved charge transport and conductivity, whereas a higher Sn-to-Pb atom ratio results in higher charge density around either Sn or Pb, resulting in a reduction in conductivity. Further increments in the Sn atoms can cause reduced device performance due to the lower orbital capacity of tin. The studies on 2D mixed Pb-Sn perovskite single crystals grown by temperature cooling crystallization observed the charge carrier mobility and trap density of 9.23 cm^2^/Vs, 1.03 × 10^9^ cm^−3^ for (C_8_H_9_F_3_N)_2_SnI_4_ single crystals, while for (C_8_H_9_F_3_N)_2_Pb_0.5_Sn_0.5_I_4_ values were 1 cm^2^/Vs, 1.22 × 10^9^ cm^−3^ [25]. From the above studies, we can infer that the optimum concentrations of mixed halides of Pb-Sn could be an ideal way to improve the charge carrier transport properties of Sn single crystals. The studies of Sn-based perovskite thin films have pointed out the issue of high p-doping due to Sn vacancy formation resulting in reduced charge carrier lifetimes and increased recombination, leading to the lattice instabilities, which eventually lead to poor device performances [63,64]. The results of the Sn single crystals have also confirmed the issue of p-doping limiting charge transport, as discussed in the section. Strategies such as the introduction of n-dopants as well as extrinsic metal doping with cations such as bismuth and scandium can help mitigate the issues of p-doping [45,65]. There are limited studies investigating the mechanisms of charge transport properties in Sn single crystals, and thus the mobility and resistivity values are not well established nor widely reproduced. Nonetheless, the reported values for mobilities are very promising in view of optoelectronic applications, and especially in solar cells and LEDs. Even if further research is clearly needed, carrier masses and intrinsic mobilities again appear not inferior in Sn perovskites than in Pb ones.

### 3.3. Defects and Doping

**Figure 4:**
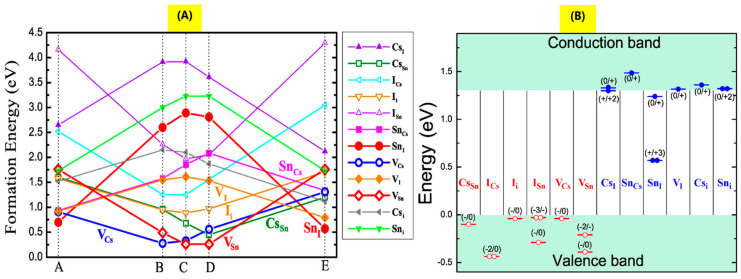
(**A**) Calculated defect formation energies in CsSnI_3_ as a function of the chemical potentials of electrons, Cs, and Sn. Points A and E correspond to Sn-rich growth conditions, while points B, C, and E correspond to Sn-poor growth conditions. Empty (solid) circles represent acceptor (donor) defects. (**B**) Calculated transition energies for various defects, with acceptor (donor) defect levels shown in red (blue). The number of empty (solid) circles denotes the number of holes (electrons) released following defect ionization. (Reproduced with permission [66], Copyright ACS).

Defects generated near the surface or the grain boundaries can result in various instability issues, which are major causes of reduced device performance. Wei et al. studied intrinsic point defects in CsSnI_3_ using first principle calculations [66]. Intrinsic defects include vacancies (V_Cs_, V_Sn_, V_I_), antisite substitution (Cs_Sn_, Sn_Cs_, Cs_I_, I_Cs_, Sn_I_, I_Sn_), and interstitial atoms (Cs_i_, Sn_i_, I_i_). They studied the defect formation energy and whether defects lead to p-doping, n-type doping, or form nonradiative recombination centers. Sn-poor and Sn-rich growth conditions were evaluated, as shown in Figure 4. Under the Sn-poor growth conditions (points B, C, and D in Figure 4A), a high concentration of V_Cs_ and V_Sn_ acceptor defects is expected due to their low formation energies. Figure 4B shows that only Sn_I_ defects can generate a charged donor state deep in the energy gap, introducing an efficient nonradiative electron–hole recombination pathway. All other defects lead to shallow acceptors and donors, or even acceptors with energies below the VBM and donors with energies above the CBM. Therefore, under Sn-poor growth conditions, V_Cs_ and V_Sn_ acceptor defects are expected to be easily ionized, leading to a high concentration of hole carriers, consistent with experimental results [67]. Moreover, Figure 4A shows that at B, C, and D points, the concentration of nonradiative recombination centers (Sn_I_) should be quite low due to their high formation energy. This allows for efficient band-to-band radiative recombination despite the high hole concentration, which agrees with experimental results [68]. Finally, Wei et al. suggested that if the growth is tuned toward Sn-rich conditions, the concentration of donor defects—specifically, Sn_I_ and V_I_—should increase, while the concentration of acceptor defects should decrease. Consequently, CsSnI_3_ crystals are expected to become almost intrinsic semiconductors due to n- and p-doping compensation, or even weakly n-doped. However, this would also activate nonradiative recombinations due to the increasing concentration of Sn_I_ defects. Similar theoretical studies were conducted for FASnI_3_ and MASnI_3_ [52]. These investigations revealed that the conductivity of FASnI_3_ can be adjusted from p-type to intrinsic by moving towards Sn-rich and I-poor growth conditions, whereas MASnI_3_ is always a p-type semiconductor with a very high hole density. These results align with experimental observations showing that FASnI_3_-based solar cells perform better [69]. The different behaviors of FASnI_3_ and MASnI_3_ were attributed to the higher formation energy of V_Sn_ acceptor defects in FASnI_3_. This is due to the weaker antibonding interaction between Sn-5s and I-5p orbitals, which is a consequence of the larger size of the FA cation and the longer Sn–I bond length in FASnI_3_.

Defects and doping densities are what primarily distinguish Sn perovskite from Pb counterparts. A review of the literature studies indicates an acute need for further research on how to reduce doping concentrations. The most obvious strategy, i.e., counterdoping, is effective in reducing background doping but has the drawback of introducing a large concentration of defects and thus reducing carrier lifetime and increasing nonradiative recombination. Future developments will therefore have to limit the doping concentration by limiting the defects that generate it. Fortunately, the piece of good news is that theoretical calculations indicate that such an avenue can be reasonable pursued since the origin of doping is not intrinsic but instead proceeds from surface defects. Sn perovskites even appear to be photostable and more defect tolerant than their Pb counterparts. The single-crystal approach to Sn perovskite fabrication appears thus as an almost ideal tool to limit doping concentrations by controlling surface reactivity while taking full advantage of the outstanding intrinsic bulk properties of Sn perovskites.

## 4. Applications of Single Crystals of Sn-Based Halide Perovskites

### 4.1. Photodetectors

**Figure 5:**
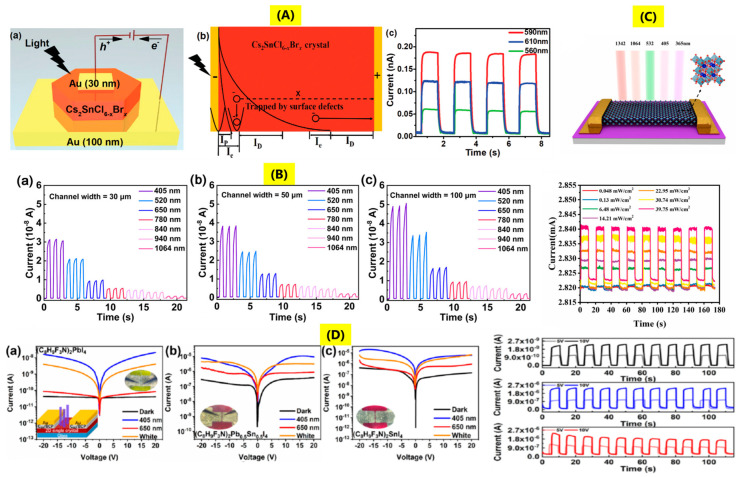
(**A**) (a) Schematic of Cs_2_SnCl_6−x_Br_x_ single crystal photodetector device, (b) mechanism of the photodetector, where I_p_, I_e_, I_D_ denote penetration, electron diffusion, and drift length; (c) current vs. time response at −20 V bias with illumination wavelengths of 590, 610, and 560 nm with light intensity of 1.3 mW/cm^2^ (reproduced with permission [28], Copyright Wiley). (**B**) (a–c) current vs. time response of (MAPbI_3_)_x_(FASnI_3_)_1−x_ (x = 0.8, 0.5, and 0.2) single crystals at 0 V bias with channel width of 30, 50, and 100 m in wavelength range of 405–1064 nm with light intensity of 0.60 mW/cm^2^ (reproduced with permission [34], Copyright ACS). (**C**) Device structure of 2D Cs_2_SnI_6_ single crystals and current vs. time response at different power densities at 1 V bias under illumination by light of wavelength 405 nm (reproduced with permission [72], Copyright Elsevier). (**D**) (a–c) current vs. voltage curves of (C_8_H_9_F_3_N)_2_Pb_1−x_Sn_x_I_4_ (x = 0, 0.5, and 1) single-crystal photodetectors, where the insets depict the device structure and optical microscope images in dark and illumination with light of wavelengths 405, 650 nm with light intensity of 0.006, 6.37, and 70.7 mW/mm^2^. The current vs. time curves were obtained at 5, 10 V bias (reproduced with permission [25], Copyright Royal Society of Chemistry).

The sensitivity of the photodetector, response speed, as well as the stability of the overall fabricated photodetector device are the key parameters that confirm the formation of a good photodetector [70]. The high absorption coefficient as well as the high carrier mobility of the perovskite, especially the decreased toxicity and environment suitability as compared to lead-based perovskites, are the key drivers for research on lead-free perovskites as promising materials for photodetector applications [71]. Z Xia et al. [28] investigated the utilization of inorganic Cs_2_SnCl_6−x_Br_x_ single crystals for narrow band photodetectors. The schematics of the photodetector device are shown in Figure 5A(a), where top and back contacts were made by thermal evaporation of gold, 30 nm and 100 nm thick. The limited thickness of the gold layer (30 nm) minimized light absorption by the metal. The light penetration length (I_p_) is proportional to the inverse of the extinction coefficient. Short-wavelength light results in high extinction coefficient and short penetration length, as shown in Figure 5A(b). Thus, the generated charge carriers are mainly near the Au-perovskite crystal surface; the photocarriers diffuse quickly within the I_e_ (electron diffusion length) and are trapped by the surface defects. On the other hand, charge carriers generated by long-wavelength light with longer penetration length are driven to positive electrodes by an applied electric field with less surface charge recombination and high collection efficiency (Figure 5A(c)). The current vs. time (I–T) photoresponse at a bias of −20 V was found to be stable and repeatable in both dark and light illumination, indicating good device stability, while the resistivity and detectivity of the device were observed to be 3 × 10^11^ Ω and 2.71 × 10^10^ Jones. The device performance was found to retain 90% of its initial response at relative humidity of ~60% for 20 days. Q Li et al. [34] explored the photodetection capabilities of hybrid mixed-halide (MAPbI_3_)_0.2_(FASnI_3_)_0.8_ single crystals. The device configuration was Au/(MAPbI_3_)_0.2_(FASnI_3_)_0.8_/Au, and it was noticed that under 0 V bias, there was production of 15 mV electric field, indicating self-driven capability. The investigation of I–T curves with different devices having a channel length of 1 mm and channel widths of 30 µm, 50 µm, and 100 µm revealed the increment of photocurrent with widening of the channel width, enlarged effective area. However, for detectors with different channel widths, there was a decrease in photocurrent with a red shift in the wavelength of light, due to the varying strength of the built-in electric fields at different incident light wavelengths ranging from 405 to 1064 nm with an intensity of 0.60 mW/cm^2^ (Figure 5B(a–c)). The responsivity and detectivity were found to be 0.247 A/W, 0.012 A/W, and 1.17 × 10^12^ Jones, 2.58 × 10^10^ Jones at 405 nm and 1064 nm. The rise and decay times of the device were found to be 59 ms and 41 ms, whereas the overall stability of the device was only 3 h, affected by the oxidation of Sn. Z Chang et al. [36] also explored the single crystals of mixed-halide (FASnI_3_)_0.1_(MAPbI_3_)_0.9_ for NIR detection. A multiple-layer photodetector was made, where PTAA (2,4,6-trimethylphenyl amine) was used as a hole transport layer and PC_61_BM (90 nm)/C_60_ (20) nm double fullerene layer as an electron transport layer were coated on patterned ITO substrate. Then, a 3 nm BCP (2,9 dimethyl-4,7-diphenyl-1,1,0-phenanthroline) layer was deposited, followed by an 80 nm silver (Ag) to get the top electrode. The device configuration is ITO/PTAA/(FASnI_3_)_0.1_(MAPbI_3_)_0.9_/PCBM/C_60_/BCP/Ag. The device area, the photocurrent, and the dark current of the device were calculated to be 6.9 × 10^−5^ cm^2^, 1.32 × 10^−8^ A, and 2.4 pA under 0 V. The low dark current resulted from the low trap density of single crystals, while the high photocurrent was due to the well-matched energy levels. The responsivity and detectivity of the device were found to be 0.53 A/W, 7.09 × 10^10^ Hz^1/2^/W, whereas the rise and fall time was 22.78 µs, 20.35 µs. Y Niu et al. [49] explored the photodetection property of 1D MDASn_2_I_6_ single crystals (MDA-4,4′ methylenedianiline). The photodetectors were fabricated by using gold electrodes with a channel width of 50 µm. The values of responsivity and detectivity at 10 V bias were found to be 4.65 mA/W and 8.85 × 10^10^ Jones. It was also noticed that an increase in the incident light intensity from 28.1, 70.25, and 140.5 mW/cm^2^ resulted in decreased values of responsivity and detectivity, which were attributed to the higher bimolecular recombination rates. Spontaneous polarization was also observed; the measured current versus voltage curves could be switched based on the polarization direction of the crystal. Current vs. time measurements for 5 on–off cycles under 0 V bias were found to exhibit good stability and reproducibility. Moreover, as the applied pressure increased from 0 GPa to 16 GPa, the photoresponse of the device was found to increase. Additionally, a reversible color change was observed, shifting from yellow to red to black and returning to the initial color when the pressure was reduced back to 0 GPa. J Xie et al. [72] studied the photodetector behavior of Cs_2_SnI_6_ single crystals. The responsivity and the detectivity of the device were found to be 6.25 × 10^5^ A/W and 1.27 × 10^11^ Jones, respectively, while the rise and fall times were 31.32 ms and 72.42 ms. The device structure and the corresponding I–T curves with different light intensities are shown in Figure 5C. The I–T curves show good reproducibility and stability for nine on–off cycles at intervals of 10 s at a bias of 1 V. X Tao et al. [25] also investigated the 2D perovskite (C_8_H_9_F_3_N)_2_Pb_1−x_Sn_x_I_4_ (x = 0, 0.5, 1) single crystals for photodetectors. The measured photocurrents are as shown in Figure 5D(a-c), where the maximum photoresponse was found at 405 nm. I–T curves also show good stability and reproducibility for 11 on–off cycles at a bias of 5, 10 V. The dark current to photocurrent ratio at 10 V was found to be higher in the case of x = 0.5 as compared to x = 1. The resistivity and detectivity of the devices were found to be 13.8 mA/W, 8.7 × 10^10^ cm Hz^1/2^/W for x = 0, 668.1 mA/W, 8.6 × 10^10^ cm Hz^1/2^/W for x = 0.5, and 32.3 mA/W, 4.7 × 10^9^ cm Hz^1/2^/W for x = 1. (C_8_H_9_F_3_N)_2_Pb_0.5_Sn_0.5_I_4_ was found to be the best device with photoresponse stability for more than 7 months at ambient conditions.

### 4.2. Solar Cells

**Figure 6:**
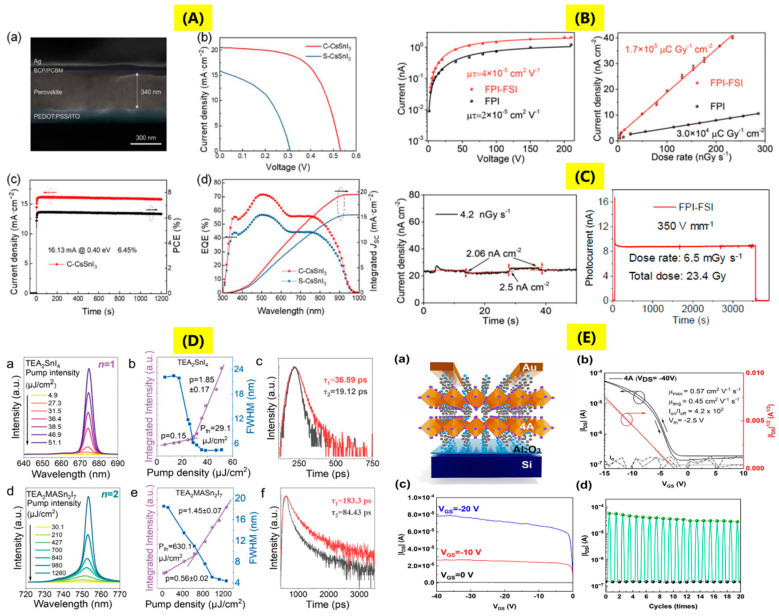
(**A**) (a) Cross-section scanning electron microscope image of C-CsSnI_3_, (b) photocurrent density vs. voltage curve, (c) stabilized power output, and (d) external quantum efficiency curves of C-CsSnI_3_, S-CsSnI_3_ perovskite solar cells (reproduced with permission [51], Copyright Royal Society of Chemistry). (**B**,**C**) Mobility-lifetime values, sensitivity, of X-ray detectors of FPEA_2_PbI_4_ and heterojunction FPEA_2_PbI_4_-FPEA_2_SnI_4_ tin single crystal, X-ray photocurrent signals, operational stability of FPEA_2_PbI_4_-FPEA_2_SnI_4_ (reproduced with permission [37], Copyright Wiley). (**D**) (a,d) Microphotoluminescence spectra, (b,e) FWHM, integrated intensity of emission peaks as function of pump density, (c,f) time-resolved photoluminescence spectra below and above pump fluence threshold of (TEA)_2_SnI_4_ and (TEA)_2_(MA)Sn_2_I_7_ tin perovskite single crystals (reproduced with permission [42], Copyright Royal Society of Chemistry). (**E**) (a) FET device structure, (b) transfer curve, (c) current vs. voltage curve, (d) on–off switching curves showing good stability of 4AMPSnI_4_ Dion–Jacobson tin perovskite single crystals (Reproduced with permission [43], Copyright ACS).

The first inorganic Sn perovskite solar cell was made by using CsSnI_3_ as a type of Schottky cell in 2012 with an efficiency of 0.99% [67]. In the year 2014, Kumar and group [73] fabricated a lead-free CsSnI_3_ perovskite solar cell with device configuration ETL/CsSnI_3_/HTL/Au (where the electron transport layer (ETL) was TiO_2_ and the hole-transporting layer (HTL) was 4,4′,4″ tris (N,N phenyl-3-methylamino-triphenylamine or Spiro-OMeTAD), where there was also the addition of different mol% of SnF_2_ to control the metallic conductivity of the CsSnI_3_ absorber. The best photovoltaic device was obtained for the case where 20% SnF_2_ was used with a measured efficiency of 2.02%. During the same year, reports by F Hao et al. [74] and N. K Noel et al. [75] observed efficiencies of 5.73% and 6.4% for CH_3_NH_3_SnI_3_ hybrid perovskite thin-film solar cells. In 2016, LJ Chen et al. observed the efficiency of around 12.96% for CsSnI_3_ perovskite quantum rods with the same charge transport layers [76]. Single crystals have been projected to exhibit improved photovoltaic performances than polycrystalline films due to lower bulk defect concentrations (~3–5 orders of magnitude), absence of grain boundaries, but still there are certain issues for higher efficiencies [18]. The thickness of the perovskite single crystals, however, plays an integral role in successful use in solar cells. It has been observed that thick single crystals can result in higher photocurrent due to below band gap absorption, but it also increases Frenkel and Schottky defects. Optimum control over thickness is therefore essential for an efficient photovoltaic device [77,78]. The typical fabrication of single-crystal perovskite solar cells can be either vertical structure (ITO or FTO/ETL or HTL/Perovskite/HTL or ETL/metal) or lateral structures (metal/perovskite/metal or metal/ETL or HTL/perovskite/metal) [18]. Q Sun et al. [51] explored the use of CsSnI_3_ perovskite single crystal for perovskite solar cell with the device configuration of ITO/PEDOT: PSS/CsSnI_3_/PC_61_BM/BCP/Ag, where PEDOT: PSS (poly(3,4-ethylenedioxythiophene) polystyrene sulfonate), BCP (bathocuproine), and PCBM ([6]-phenyl C_61_ butyric acid methyl ester). In this work, two different devices were investigated, where the device obtained after precursor engineering with CsSnI_3_ single crystal was denoted as C-CsSnI_3_, while the device made using a salt precursor of CsSnI_3_ was denoted as S-CsSnI_3_ (Figure 6A). The fabricated device using C-CsSnI_3_ achieved an efficiency of 6.53% with values of V_OC_-0.53 V, J_SC_-20.50 mA/cm^2^, and FF-0.601, and showed good stability with 91% retention of efficiency after continuous operation in N_2_ atmosphere for 1000 h. The fabricated device using S-CsSnI_3_ showed efficiency of only 2.26%, V_OC_-0.31 V, J_SC_-15.84 mA/cm^2^, and 85% retention of efficiency after continuous operation in N_2_ atmosphere for 1000 h. The better C-CsSnI_3_ device performance is due to better control over crystallization kinetics of CsSnI_3_ by using single crystal precursor engineering, thereby causing reduction in charge recombination. The external quantum efficiency was also improved in the case of C-CsSnI_3_ within the absorbance range of 300–950 nm, as seen from Figure 6A.

### 4.3. X-ray Detectors

The key parameters for a good X-ray detector are X-ray attenuation ability—higher the attenuation of X-ray, greater interaction of high-energy photons, smaller the thickness of material required, charge carrier mobility (µ), carrier lifetime (τ), product of µτ, sensitivity—charge collected per unit area under X-ray exposure; high sensitivity means higher photocurrent at a particular radiation dose; limit of detection—it determines the minimum detection of the X-ray radiation of the detectors. Ideally, its value should be low to reduce ionizing radiation; light yield; afterglow; and emission wavelength also play a key role [79]. M Li et al. [37] explored the X-ray detection application of heterojunction of the FPEA_2_PbI_4_-FPEA_2_SnI_4_ single crystal. The synthesis process began with the first preparation of FPEA_2_PbI_4_ and FPEA_2_SnI_4_ single crystals; thereafter, to make the heterojunction, FPEA_2_SnI_4_ single crystal was placed in FPEA_2_PbI_4_ solution, wherein growth of FPEA_2_PbI_4_ slowly progresses on FPEA_2_SnI_4_ by solvent evaporation. For X-ray detectors, electron transport layers C_60_ (30 nm) and BCP (8 nm) were deposited on the FPEA_2_SnI_4_ by thermal evaporation, then 30 nm Au cathode, while 30 nm Cr anode was deposited on the FPEA_2_SnI_4_. Metal electrodes were kept parallel to each other. The device exhibited resistivity of 7 × 10^9^ Ω cm, a small dark current at high voltage, and a product of µτ was found to be 4 × 10^−5^ cm^2^/Vs, which was two times higher than a pure Pb-based single crystal. The detection sensitivity was found to be 1.7 × 10^5^ Gy^−1^/cm^2^, with a low detection dose rate. The device was found to be stable for 1 h under irradiation with an intensity of 350 V/mm and a high dose rate of 6.5 mG_y_/s, with no change in the original signal current (Figure 6B,C).

### 4.4. Miscellaneous Applications

Sn-based perovskite single crystals have also been utilized in some other applications, such as amplified spontaneous emission, second harmonic generation, nonlinear optical switching, and field effect transistors (FETs). Two-dimensional Ruddlesden–Popper (TEA)_2_(MA)_n-1_Sn_n_I_3n+1_ (n = 1, 2, TEA, MA is 2-thiophenethylamine, methylamine) single crystals were explored for amplified spontaneous emission [42]. The pump fluence-dependent amplified spontaneous emission of exfoliated (TEA)_2_SnI_4_, (TEA)_2_(MA)Sn_2_I_7_ single crystals was determined at a temperature of 20 K by irradiation of the samples using a laser beam of wavelength 400 nm with a pulse rate of 150 fs. The microphotoluminescence spectra of (TEA)_2_SnI_4_, (TEA)_2_(MA)Sn_2_I_7_ single crystals as a function of pump fluence can be seen from Figure 6D(a,d). At power density (P) = 4.9 μJ/cm^2^, (TEA)_2_SnI_4_ shows broad emission at 674 nm with FWHM~22.13 nm. When the value of P exceeds the threshold, there is an increase in intensity and a decrease in FWHM to 4.41 nm of the photoluminescence spectra (Figure 6D(b)). The threshold value was observed at 29.1 μJ/cm^2^, and the photoluminescence lifetime at low excitation was 36.89 ps, while at high excitation, the lifetime reduced to 19.12 ps (Figure 6D(c)), which depicts a population inversion resulting in a stimulated emission. In the case of (TEA)_2_(MA)Sn_2_I_7_, FWHM varied from 18.52 nm to 4.4 nm with increasing P and a threshold value of 630.1 μJ/cm^2^ (Figure 6D(e)). The lifetimes below and above the threshold were 183.3 ps and 84.43 ps (Figure 6D(f)). The increased value of the threshold for (TEA)_2_(MA)Sn_2_I_7_ could be due to the size or thickness of the single crystal. The second harmonic generation (SHG) is a common nonlinear optic effect; the key requirement for an SHG material is that the crystal structure should be non-centrosymmetric. Nonlinear optical materials generate SHG when stimulated by an optical field, which have received considerable attention for laser devices. K Tao et al. [50] explored 1D Sn-based chiral perovskite single crystals for second harmonic generation. Chiral hybrid perovskite single crystals of (R/S-α-PEA)SnCl_3_ and (R/S-α-PEA)SnBr_3_ were developed using organic ions R, S-α-phenylethylammonium (R/S-α-PEA^+^). For measuring the second harmonic generation, synthesized crystals were dispersed on a glass substrate, while the SHG signals were studied with the help of a microscope with a femtosecond laser source. The wavelength-dependent SHG spectra of (S-α-PEA)SnCl_3_ and (S-α-PEA)SnBr_3_ show narrow, sharp peaks when excited with various excitation wavelengths ranging from 1020 nm to 900 nm with a power of 10 mW. The emission wavelength is found to be just half of the incident wavelength: 510 to 450 nm. The power-dependent SHG spectra with an excitation wavelength of 1020 nm were studied, and it was observed that an increase in the incident laser power results in increased SHG signals. SHG properties were also compared to Y-cut α-quartz; (S-α-PEA)SnBr_3_ showed SHG response of 1.4 x Y-cut α-quartz, which was higher than (S-a-PEA)SnCl_3_-0.9 x Y-cut α-quartz. Such a difference is attributed to the different band structures, as SHG is dependent on valence and conduction band. Another promising application of halide perovskites is the nonlinear optical switches undergoing a reversible transformation among SHG-ON and SHG-OFF states in the presence of external signals, which show good promise for optical communications, photonics, and data memories [80,81]. Kai Li et al. [44] explored the prospect of multilayered tin-based perovskite single crystals of (BA)_2_(EA)_2_Sn_3_Br_10_ (where BA and EA are butylammonium and ethylammonium) for nonlinear optical switching applications. The SHG responses of the single crystal were measured using an Nd:YAG laser of wavelength 1064 nm with a 5 ns pulse duration in the 300–375 K temperature range. At low temperatures, the crystal showed SHG-ON state; an increase in the temperature resulted in a reduction in the SHG signal close to the phase transition temperature (353 K); after this temperature, the crystal showed negligible signal; it reaches SHG-OFF state. The SHG switching signal contrast was estimated by the ratio of intensity between the SHG-ON and SHG-OFF states, and the value was found to be 35. The reason for switching characteristics was attributed to the large EA cation causing octahedral distortions and order–disorder transformation of organic cations. C Liao et al. [43] explored 4AMPSnI_4_ (AMP denotes (aminomethyl)-piperidinium), a Dion–Jacobson tin perovskite single crystal for field effect transistors. The first step in preparation of the FET was coating n-doped silicon substrates with ~60 nm Al_2_O_3_ by atomic layer deposition. Two devices were fabricated, one was 4AMPSnI_4_ and the other was PEA_2_SnI_4_ (PEA-phenylethylammonium). The single crystals were dissolved in DMSO and DMF, followed by spin-coating and annealing at 100 °C for 15 min. In the last step, a 60 nm gold electrode via thermal evaporation was deposited. The device configuration was Si/Al_2_O_3_/4AMPSnI_4_ or PEA_2_SnI_4_/Au with channel length 50 µm, width 1000 μm. The typical device structure of 4AMPSnI_4_ and its various device characteristics are as shown in Figure 6E(a-d). The hole transfer characteristics of the device (Figure 6E(b,c)) were measured at V_DS_ (drain voltage) of −40 V and V_GS_ (gate voltage scanning) from 10 to −15 V at ambient conditions. There was observation of small hysteresis but no ion migration due to negligible grain boundaries. The threshold voltage was at −2.5 V, and the device exhibited p-type modulated conduction with average and maximum hole mobilities of 0.45 and 0.57 cm^2^/Vs. The on–off ratio was 4.2 × 10^2^ and good cycling stability for 20 on–off cycles (Figure 6E(d)). In the case of PEA_2_SnI_4_ FET, the threshold voltage was 0.9 V, and there was observation of maximum hole mobility to be 0.39 cm^2^/Vs and a high on–off ratio of 1.4 × 10^3^. 

## 5. Current Challenges, Conclusions and Assessments

The review article explored the development of single crystals of Sn halide perovskites by providing information about various synthesis methods, optical and electrical properties, defects, and their prospects for use in applications such as perovskite solar cells, photodetectors, X-ray detectors, etc. The motivation for Sn-based perovskites is to combine the photoconversion efficiencies demonstrated for perovskite solar with lead-free, sustainable materials. Life-cycle analysis studies have compared the environmental impact of lead and tin-based perovskite solar cells from cradle to grave, considering the effects of solvents, heavy metal poisoning, and end-of-life disposal of photovoltaic devices, and established tin-based perovskites as a much more benign option [82,83,84,85,86,87,88]. The main driver for research on single crystal perovskites is the absence of grain boundaries, lower trap density, and higher mobility with respect to the standard choice of polycrystalline films. Such advantages are especially important for Sn perovskites, as the perovskite surface is very quickly degraded when exposed to ambient oxygen and water, and therefore the absence of grain boundaries makes the perovskite passivation much more efficient. Furthermore, once the surface has been passivated, calculations show that Sn-based perovskites are not photoactive and bulk crystals do not tend to degrade under illumination, a very promising feature to realize robust solar cells. Such clear advantages of Sn-based single crystal perovskites are compounded by the few key challenges that need to be overcome for their successful usage in optoelectronic applications. 

The main issue outlined in this review is the challenge of growing single crystals thin enough to be used in solar cells. A universal growth method for forming uniform, high-quality, and most importantly, thickness-controlled single crystals is still lacking. Moreover, another fabrication issue that needs to be resolved is the control of lateral size, also minimizing growth time and defect density while maximizing homogeneity. Low-temperature methods, the use of green solvents, and modification of the well-known synthesis methods could improve single crystal growth with a well-controlled structure. Another issue is the stability and oxidation of Sn under ambient conditions. On one hand, proper encapsulation and surface heterostructures are approaches that are being exploited with increasing success. Nevertheless, some degree of oxidation is always present, causing typical p-type background doping. This needs to be counterbalanced with controlled n-type doping and the use of polymers or other additives. Various strategies, such as using reducing agents, controlling crystallization, and partial ion substitution, have already been proposed. Future breakthroughs may come from finding stable solvents, improving our understanding of the Sn chemistry, and possibly employing the vacuum-deposited technique to address solubility issues. While vacuum deposition is currently well established for producing high-quality polycrystalline films, future advancements in single crystal growth may necessitate the use of patterned substrates or additional single crystals as substrates to facilitate epitaxy. Emerging methods are focusing on growing halide perovskites using vacuum deposition techniques with precise control over crystal orientation [89]. This approach integrates sophisticated deposition techniques with advanced substrate engineering. Future developments may involve creating substrates with innovative patterns to precisely guide crystal nucleation and growth. Additionally, advancements in substrate surface treatments and coatings could enhance the adhesion and control of crystal orientation.

In conclusion, single crystals of Sn-based lead-free halide perovskites show good prospects for replacing toxic Pb-based halide perovskites in solar cells as well as in other applications, such as light-emitting diodes, optical communications, photocatalysis, and sensing. The combination of theoretical and experimental works highlights the basic research directions that need to be addressed to improve performance, reproducibility, and stability of Sn-based single crystal perovskites to achieve devices that are robust and efficient enough to be commercialized.

## Figures and Tables

**Table 1:** The band gap variation in various single crystals of Sn-based halide perovskites.

Single Crystal Sn Perovskites	Band Gap (eV)	References
CsSnI_3_ (γ-phase)	1.3	[60]
CH_3_NH_3_SnI_3_, CH(NH_2_)_2_SnI_3_	1.15, 1.4	[26]
NH(CH_3_)_3_SnCl_3_, NH(CH_3_)_3_SnBr_3_	3.59, 2.76	[47]
MASnBr_3_ MAPb_0.74_Sn_0.26_Br_3_, MAPb_0.68_Sn_0.32_Br_3_, MAPb_0.39_Sn_0.61_Br_3_	2.02 1.88 to 1.77	[27]
DMASnI_3_ (DMA-CH_3_NH_2_CH_3_^+^)	2.48 to 1.32	[24]
Cs_2_SnCl_6−x_Br_x_ (x-0 to 6)	4.66 to 3.34 (x-0 to 6)	[28]
PEA_2_SnBr_4_ Bi doped PEA_2_SnBr_4_ (0 to 20%)	2.60 2.63 to 2.59	[45]
CsSn_x_Pb_1−x_Br_3_ (0 ≤ x ≤ 1)	1.74 to 1.62	[29]
FASnI_3_	1.25	[61]
Tetragonal MASnBr_3_	2.07	[48]
(FASnI_3_)_0.1_(MAPbI_3_)_0.9_	1.35	[36]
Cs(Pb_0.75_Sn_0.25_)(Br_1.00_Cl_2.00_)	1.84 to 2.42	[31]
(C_8_H_9_F_3_N)_2_Pb_0.5_Sn_0.5_I_4_, (C_8_H_9_F_3_N)_2_SnI_4_	1.94, 2.02	[25]
(C_13_H_16_N_2_)Sn_2_I_6_	2.61	[49]
(R/S-*α*-PEA)SnCl_3_, (R/S-*α*-PEA)SnBr_3_	3.70, 3.23	[50]

## Data Availability

The relevant data are contained in the article.
